# Bladder Neck Incision in Posterior Urethral Valve Management: a Meta-Analysis with Insights into Adjunctive Bladder Interventions

**DOI:** 10.1007/s11934-025-01309-w

**Published:** 2025-12-23

**Authors:** Mohamed Tharwat, Reham Ramadan, Abdelwahab Hashem, Diaa-Eldin Taha, Mohamed Hussiny, Ahmed Elkashef, Tamer E. Helmy, Mohamed S. Dawaba, Ashraf T. Hafez

**Affiliations:** 1 Urology Department, King Salman Armed Forces Hospital, Tabuk city, Kingdom of Saudi Arabia; 2 Egyptian Ministry of Health, Dakahlia, Egypt; 3 Urology department, Faculty of medicine, Delta University for Science and Technology, Dakahlia, Egypt; 4 Urology department, Faculty of medicine, Kafrelsheikh University, Kafrelsheikh, Egypt; 5 Pediatric Urology department, Urology and Nephrology Center, Mansoura, Egypt

**Keywords:** Posterior urethral valve, Alpha blockers, Voiding dysfunction, Valve ablation

## Abstract

**Purpose of Review:**

To evaluate the outcomes of bladder neck incision (BNI) and alternative treatments, such as alpha blockers, in improving voiding dysfunction in posterior urethral valve (PUV) after valve ablation (VA).

**Recent Findings:**

Eleven studies comprising 826 patients with PUV were included. Concomitant BNI and VA significantly improved both VUR resolution (Risk Ratio “RR”= 1.36; 95% CI: 1.04–1.79), and postoperative serum creatinine -0.16 (95% CI: -0.28 to -0.04). Concomitant BNI and VA significantly improved detrusor overactivity (RR = 0.44; 95% CI: 0.22–0.91; p = 0.03), and reduced significantly maximum detrusor pressure at Qmax (Pdetmax) by-23.53 (95% CI: -35.01 to -12.05). It also significantly decreased both the use of alpha blocker/anticholinergic (RR = 0.59; 95% CI: 0.36 to 0.95), and the need for intermittent catheterization (RR = 0.16; 95% CI: 0.03 to 0.83; p = 0.03). However, there were no significant difference either in bladder compliance (RR = 0.75; 95% CI: 0.31 to 1.79), nor children required re-intervention (RR = 0.52; 95% CI: 0.25 to 1.05). Alpha blockers after VA showed a pooled post-void residual urine reduction of -59.44 (95% CI: -129.05 to 10.17). The pooled estimate for peak flow rate with alpha blockers was 14.22 (95% CI: 13.70–14.74).

**Summary:**

BNI combined with valve ablation could improves bladder function and reduces the need for additional interventions compared to VA alone.

**Supplementary Information:**

The online version contains supplementary material available at 10.1007/s11934-025-01309-w.

## Introduction

Posterior urethral valves (PUV) represent the primary etiology of congenital obstruction within the urethra in male neonates, occurring in 2.6 per 10,000 births [1], which is responsible for 16.8% of renal impairment in pediatric patients with end-stage renal disease (ESRD) [2].

A significant portion of PUV patients have some basal renal functional impairment.The primary aims in the diagnosis and management of PUV is to prevent more acquired renal damage, and to improve bladder function. Although valve ablation (VA) remains the mainstay of initial management of PUV to resolve urinary obstruction, valve bladder syndrome (VBS) defined as abnormalities in bladder storage and emptying could lead to upper tract dysfunction and renal impairment occurred even after VA and was first described by Mitchell in 1982 [1, 3].

Bladder storage dysfunction results from impaired bladder compliance often associated with detrusor overactivity, while bladder emptying dysfunction is commonly due to bladder neck hypertrophy [3–5]. Bladder outlet obstruction is most often primary caused by PUV, with secondary bladder neck hypertrophy as a response of the bladder muscle to the obstructed valve.

Bladder outlet obstruction after VA can be secondary to bladder neck obstruction, residual PUV, or urethral stricture. Secondary bladder neck obstruction can be defined as increased voiding detrusor pressure (Pdet) and obstructed uroflow in the absence of residual PUV and urethral stricture. Bladder neck hypertrophy can affect voiding, storage, and renal function [1, 4, 5].

Following VA, the primary aim of therapy in VBS is to enhance bladder emptying and lower storage pressures to near-normal levels, thereby reducing stress on the upper urinary tract [5]. Various adjuvant treatments for bladder neck hypertrophy are proposed including medications like alpha blockers, bladder neck Botox injections, bladder neck incision (BNI) [6–8].

Concomitant BNI intervention and VA in the management of PUV remains debated. While some studies report significant improvements with this combined approach, others find no measurable benefit compared to valve ablation alone [7, 9–12] Furthermore, alternative strategies, such as alpha-blocker therapy and Botox injections at the bladder neck, have also been explored with mixed results [6, 13–17].

In this systematic review and meta-analysis, we aim to assess the outcomes of bladder neck interventions in patients with PUV, focusing primarily on BNI. We also evaluate the effectiveness of other treatments, such as alpha-adrenergic blockers, focusing on their impact on bladder function, voiding dysfunction, and kidney outcomes. We seek to answer the question: Do these interventions improve bladder and renal outcomes in patients with PUV? This analysis aims to provide a clearer understanding of their role in managing bladder neck complications and improving patient outcomes, ultimately guiding future therapeutic decisions.

## Materials and methods

### Study Design

This systematic review and meta-analysis followed the updated PRISMA guidelines and the Cochrane Handbook for Systematic Reviews of Interventions [18, 19]. The systematic review was listed in PROSPERO (ID: CRD42024616979), the global registry for systematic reviews managed by the National Institute for Health Research.

We carried out an extensive search across major databases, including PubMed, Web of Science, Scopus, and the Cochrane Library. By November 2024, we had used carefully chosen keywords that aligned with our research focus. We applied no limitations when searching the databases. The full search details can be found in Supplementary File 1.

#### Inclusion Criteria

This review includes studies involving patients diagnosed with posterior urethral valves (PUV) who underwent treatment for bladder outlet obstruction. Studies were included if they evaluated interventions involving bladder neck incision (BNI), alpha-blockers, or Botox injections at the bladder neck.

The review included studies comparing one or more interventions of interest to VA or other interventions, as well as single-arm designs from which the relevant intervention arms were analyzed. The outcomes assessed in this review include vesicoureteral reflux resolution (VUR), postoperative serum creatinine levels, detrusor overactivity (DOA), maximum detrusor pressure at Qmax (Pdetmax), myogenic failure, peak flow rate (Qmax) and poor compliance with treatment. The number of patients who required re-intervention, clean intermittent catheterization (CIC), use of alpha-adrenergic blockers and anticholinergic were also assessed.

We considered all original studies, such as randomized trials, cohort studies, and non-randomized trials, without any restrictions on when they were published, to capture all relevant research.

### Exclusion Criteria

1- Studies that lack sufficient data for extraction and analysis. 2- Review papers, books, editorials, thesis, commentaries, and abstracts from conferences. 3- Articles not published in English. 4- Studies involving interventions other than valve ablation, bladder neck incision (BNI), alpha-adrenergic blockers, and Botox therapy at the BN will be excluded from this review. 4- Studies focusing solely on urinary diversion (e.g., vesicostomy, catheteriazable channels) or redo valve ablation were excluded unless they also evaluated BNI, alpha-blockers, or Botox.

Two authors first reviewed the titles and abstracts of the studies, followed by a full-text assessment of the relevant ones using an Excel sheet to check their eligibility. A third author was consulted to resolve any disagreements.

### Date Extraction and Study Outcomes

Data from the relevant studies were independently extracted by two authors using a predefined Excel sheet, which included sections for summaries, baseline characteristics, and outcomes. The summary sheet captured details such as the study ID, location, study period, design, sample size, criteria for inclusion and exclusion, Bladder Neck Interventions group, Valve ablation group, results, and follow-up information.

The baseline table included details such as the study ID, patient count, their ages, baseline serum creatinine, baseline VUR, baseline hydronephrosis, Preoperative Pdetmax, baseline DOA, baseline PVR, baseline myogenic failure, and baseline bladder hypercontractility. The parameters of the outcomes sheet were the sample size, last follow-up serum creatinine, VUR resolution, postoperative DOA, change in P detmax, number of patients who required re-intervention, number of patients who required alpha blocker/anticholinergic, number of patients who required CIC, postoperative PVR, myogenic failure, peak flow rate (Qmax), and poor bladder compliance.

Data extraction discrepancies were addressed by author discussions. We utilized certain Statistical methods to calculate the standard deviations from the p-value, median, and ranges for studies with continuous data [20, 21]. The means and standard deviations of the two groups were then combined for analysis [19].

### Outcomes Definition

All outcome definitions, including detrusor overactivity (DOA), myogenic failure, bladder hypercontractility, and bladder compliance, were based on urodynamic assessments as reported in the included studies. Specifically, DOA was defined as any involuntary bladder contraction, regardless of its intensity, that leads to incontinence or strong urgency, or as an unconstrained contraction exceeding 10 cm H₂O during the filling phase, regardless of whether leakage occurred [9, 11, 12, 22]. According to International Children’s Continence Society standardization, In children ≤ 6 years, a repetitive PVR of >20 ml or >10% bladder capacity is considered elevated. Expected bladder capacity (EBC) = age (years) × 30 + 30 (expressed in ml) [23].

Myogenic failure was identified by a combination of factors: an enlarged bladder capacity, weak bladder contractions with a detrusor pressure below 20 cm H₂O during voiding, an inability to maintain a steady contraction, and a residual urine volume greater than 10% of the bladder’s total capacity [9, 11, 12, 24].

Bladder hypercontractility was defined as the presence of uninhibited detrusor contractions or a maximum detrusor pressure (Pdetmax) exceeding 90 cm H₂O [10, 12, 25]. Bladder compliance was evaluated at 25%, 50%, 75%, and 100% of the patient’s bladder capacity. Detrusor compliance was graded using a modified version of the Khoury et al. system, categorized as severely impaired (≤ 10 ml/cm H₂O), moderately impaired (10–20 ml/cm H₂O), mildly impaired (20–30 ml/cm H₂O), and normal (>30 ml/cm H₂O). Additionally, the detrusor pressure at the end of the filling phase, known as the end infusion pressure (EIP), was measured. An EIP exceeding 20 cm H₂O was considered elevated and served as an indicator of poor bladder compliance [9, 11, 26].

### Assessment of Bias and Quality of Included Studies

Two reviewers independently assessed bias risk using the Cochrane Risk of Bias Tool for Randomized Trials (ROB 2) [27]. This comprehensive evaluation considered six key areas: randomization methods, adherence to assigned interventions, management of missing data, accuracy of outcome measurements, selection of reported outcomes, and any additional sources of bias. Reviewers categorized their judgments into “yes,” “probably yes,” “probably no,” or “no information.” [27]. The NIH tool was used to assess study quality, including parameters like study design, sample size, outcome measures, and potential risks of bias, such as selection, performance, detection, attrition, and reporting biases [28]. Any disagreements were resolved through discussion.

### Data Analysis

Statistical analysis for the double-arm studies was performed using RevMan software version 5.4.1. In this meta-analysis, risk ratios (RR) were calculated for categorical outcomes, and mean differences (MD) for continuous outcomes, with 95% confidence intervals (CIs) provided. A p-value below 0.05 was regarded statistically significant. Single-arm analysis was conducted using OpenMeta [Analyst]. This analysis calculated proportions or rates for categorical outcomes and pooled effect sizes for continuous outcomes, each presented with 95% confidence intervals (CIs).

Heterogeneity was assessed using the Chi-square Q test, with a p-value of less than 0.1 indicating significant heterogeneity. For the single-arm analysis, a random-effects model was used to account for variability across studies. In the presence of heterogeneity, a leave-one-out sensitivity analysis was performed to evaluate the robustness of the results.

## Results

### Data Collection and Study Selection

**Fig. 1:**
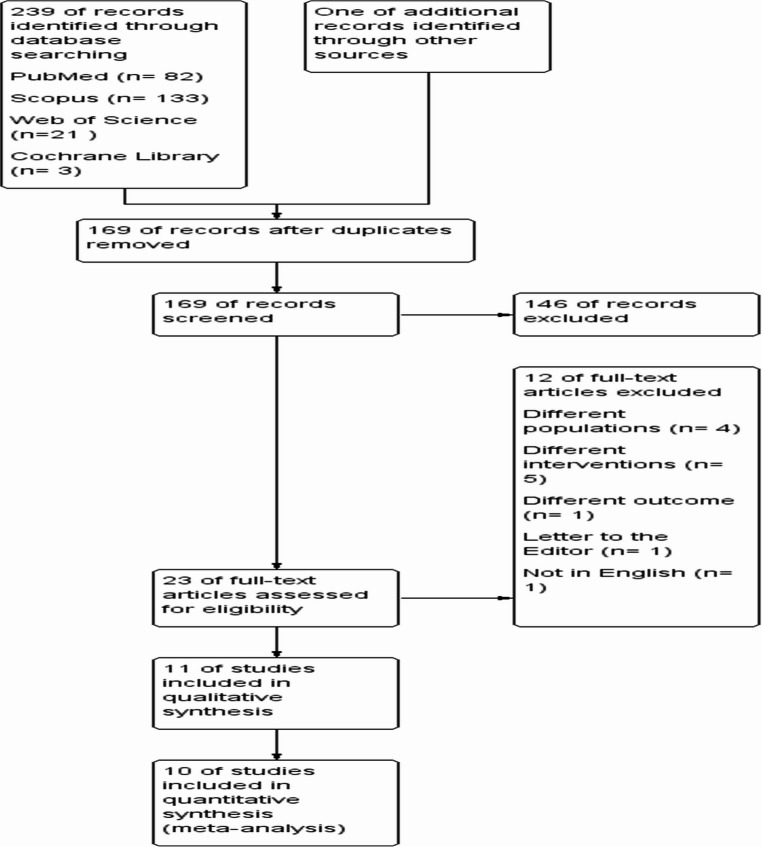
PRISMA Flow Diagram illustrating the study selection process, including records identified, screened, assessed for eligibility, and included in the final analysis

The electronic database search returned a total of 239 articles, with one additional study found through manual searching. After eliminating duplicates, 168 unique articles were left for the initial screening of titles and abstracts. Following this step, 23 articles were shortlisted for a detailed full-text review. We excluded four studies due to different populations and five others because they investigated different interventions. One study was excluded because it reported outcomes limited to treatment pattern scores and progression to end-stage bladder rather than the predefined urodynamic and functional endpoints of this review, another was a letter to the editor, and one was excluded as it was not in English. The excluded interventions included technical variations of valve ablation only, urinary diversion procedures, broad management protocols, adjunctive procedures such as ureterostomy, and alternative pharmacologic therapy (Fig. 1).

This systematic review included 11 studies with 826 patients with PUV, of whom 410 underwent valve ablation with BNI, 202 underwent valve ablation alone, 204 received alpha-adrenergic blockers, and 10 were treated with Botox injections at the bladder neck.

Across the included studies, BNI was performed as an additional single deep incision proximal to the verumontanum, with variations in site and instrument. Abdelhalim et al. used a cold knife at 6 o’clock, Singh et al. at 7 o’clock, Kajbafzadeh et al. at 6 o’clock with care to leave the adventitia untouched, Sarin et al. applied a modified technique with a hook electrode at 6 o’clock, and Sobhani et al. performed a coagulation-mode incision at 6 o’clock with precautions to avoid adventitial or verumontanum injury [7, 9–12]. Despite these technical differences, the fundamental principle across all studies was the same: a single deep incision proximal to the verumontanum. These details are summarized in Table 1. (Supplementary file 2).

**Table 1:** Summary of the included studies

Study ID	Country, and time of realization	Study design	Sample size	Inclusion criteria	Exclusion criteria	Intervention group	Valve ablation only group	Result	Follow up
Abdelhalim et al. 2022 [7]	Egypt, between January 2000 and December 2017.	A retrospective study	114	• Infants (younger than 2 years of age) diagnosed with and treated for PUV between January 2000 and December 2017. • Only patients with at least 18 months of follow-up were included.	• Patients with other anomalies that could potentially affect the lower urinary tract function. • Those with history of urinary diversion (e.g., vesicostomy or ureterostomy) were excluded.	• BNI was carried out with an additional single incision also using cold knife at 6 o’clock position stopping proximal to the verumontanum. • Adequacy of BNI was confirmed by the ability to visualize the bladder lumen with the tip of the scope at the level of the verumontanum and the expression of an adequate urine stream with Créde manoeuvre.	PVA was routinely performed using cold knife urethrotome at 12, 5 and 7 o’clock. If deemed necessary by the operating surgeon.	• Between 2000 and 2017, a total of 114 patients underwent PVA and met the study criteria with a median follow-up of 58 (18–230) months. • For group 1, 16 (22.9%) patients needed readmission. • Check cystoscopy was free and no further intervention was performed in 5(7.5%) and re-ablation was performed in 11(15.7%) patients. • For group 2, 3(14.3%) patients needed reintervention. Re-ablation and re-ablation plus BNI were performed in 1(4.8%) and 2(9.5%), respectively. • For group 3, cystoscopy was free in 1(4.3%), re-ablation and re-ablation plus BNI were performed 2(8.7%) and 1(4.3%), respectively. • There were no significant differences in the re-admission and re-intervention rates among the three study groups (*p* = 0.65 and *p* = 0.50, respectively).	Median follow-up was 58 (18–230) months
Singh et al. 2019 [9]	India, between July 2010 and July 2016.	Randomized clinical trail	71	Patients diagnosed with posterior urethral valves (PUV).	The presence of simultaneous urogenital anomalies, any neurological condition, history of any urethral manipulation, and urinary diversion	• The bladder neck incision was given at 7 o’clock position. • The incision was deep up to bladder neck muscle and from bladder neck to just before verumontanum. Catheter was removed on 3rd postoperative day.	Valve fulguration was done with right-angled electrode	• The mean age was 7.26 years in Group I and 7.66 years in Group II at the end of follow-up. • There was no statistically significant difference found regarding detrusor overactivity (*P* = 0.68), compliance (*P* = 0.052), end-filling pressure (*P* = 0.08), and max Pdet at Qmax (*P* = 0.08) in the both groups. • However, there was a statistically significant difference regarding improvement of peak flow (*P* = 0.038) and postvoid residue (PVR) (*P* = 0.045) in Group I in comparison to Group II.	Mean of 59.5 months
Sarin et al. 2013 [11]	India, between August 2009 and July 2010)	Pilot study	18	All patients with a diagnosis of PUV on micturating cystourethrogram (MCUG) and simultaneous evidence of bladder outlet obstruction were included in the study.	Those patients who had simultaneous urogenital anomalies other than PUV, history of any previous endoscopic intervention, prior vesicostomy, history of therapy with drugs that act on bladder and those who refused consent for BNI were excluded from the study.	• In children assigned to PEVI with BNI, an additional single incision was made with the hook electrode at the 6 o’clock position through the bladder neck, just proximal to the verumontanum (modified technique). • The end point was reached when it was possible to enter the bladder straight instead of the excessive upward angulation of cystoscope that was required before the procedure.	Valves were incised using a hook electrode and through an appropriate sized urethrocystoscope using pure cutting electrocautery.	• The incidence of bladder dysfunction in the two groups was similar—55.5% in case group and 66.6% in control group. • Hypocompliant, high-pressure bladder was the predominant cystometric finding in both groups. • Three patients in the case group and two patients in the control group had high end infusion pressure (EIP) with poor compliance. • Detrusor overactivity (DOA) was seen in 23.1% patients in the case group as compared to 55.5% patients in the control group (*P* = 0.3348). • Five patients in both groups were later started on anticholinergics due to raised EIP, small capacity bladder and/or DOA.	3 months
Kajbafzadeh et al. 2007 [10]	Iran, between March 1998 and April 2003	A retrospective study	46	• The study included patients with a confirmed diagnosis of posterior urethral valves (PUV) who underwent either valve ablation with bladder neck incision (BNI) or conventional valve ablation. • For comparison, an age-matched cohort of children treated with conventional valve ablation was selected.	The exclusion criteria for both groups were presence of simultaneous urogenital anomalies, history of any urethral manipulation and urinary diversion.	In children assigned to valve ablation with BNI, a single 6 o’clock incision was made just proximal to the verumontanum, carefully sparing the adventitia. The incision was performed with cutting current and hand maneuvers, with coagulating current used as needed.	Valves were incised using a hook electrode and through an appropriate sized urethrocystoscope using pure cutting electrocautery.	• Mean patient age at presentation was 1.6 years in group 1 and 1.8 years in group 2. Preoperatively, all patients in both groups had hypercontractile bladders and comparable high maximum voiding detrusor pressures. • At the end of follow up (mean 4.5 years) no patient in group 1 had bladder hypercontractility or detrusor overactivity, and the mean maximum voiding detrusor pressure was 53 ± 15 cm H2O. • In comparison, 9 patients in group 2 had bladder hypercontractility, 6 had detrusor overactivity and the mean maximum voiding detrusor pressure was 87 ± 45 cm H2O (*p* > 0.01). • Myogenic bladder failure developed in 5 patients in group 2. The number of patients requiring anticholinergic medication and the duration of treatment were also significantly higher in group 2 compared to group 1	Mean of 54 months.
Sobhani et al. 2024 [13]	Iran, between March 1998 and April 2003.	Retrospective study	301	The inclusion criteria of the study were typical PUV on VCUG as well as fibrotic high ride and hypertrophied bladder neck on video urethrocystoscopy, urodynamic evaluations before and after surgery, presence of regularly based follow-ups on urinary tract ultrasonography, serum biochemical assessments, urinalysis, and urine culture.	The exclusion criteria in this study were patients with simultaneous PUV and urogenital anomalies including duplicated urethra, anterior urethral valve (AUV), anterior urethral diverticulum (AUD) vertebral anomalies, megaureter as well as patients who lost follow-up or had history of previous endoscopic or open surgical intervention prior to have been referring to the senior author’s (AMK) clinic in the tertiary pediatric center.	• In all patients, the valves were fulgurated using a blunt tipped fexible Bagbee™ fulgurating electrode with electrocoagulation of a proper-sized neonate urethrocystoscope (4.5–6 Fr. tip with 3 Fr. working channel) (Richard Wolf^®^ Company). • All patients underwent valve ablation along with BNI via a single deep incision at the 6 o’clock position proximal to the verumontanum precisely to prevent damage to the prostate capsule; the verumontanum (located in the posterior aspect of the prostatic urethra) remained untouched. • This procedure was performed with vigilance to leave the adventitia undamaged. • The valve fulguration was done with electrocoagulation with minimal electric current. Video-endoscopic valve fulguration was per formed with 10–15% electric energy of the cautery transferred by 3 Fr. Bagbee/metal stylet and follower of 3 Fr. JJ stent. The bladder neck incision technique by coagulation mood.	*	• Mean age at diagnosis was 7.22 ± 2.45 months (ranging from 7 days to 15 months) with a mean follow-up of 5.12 ± 2.80 years. • The incidence of hydronephrosis was decreased from 266 (88.3%) at the baseline to 73 (24.3%) patients in long-term follow-up. At baseline, 188 (62.5%) patients were diagnosed with VUR, which decreased to 20 (6.6%) individuals at the end of follow-up. • Bladder and renal function were improved in follow-ups following concomitant PUV ablation and BNI. • No Myogenic failure was depicted in all patients with BNH. No ureteric reimplantation was needed during the two decades follow-up.	Mean of 61.44 months
Aboulela et al. 2024 [14]	Egypt, between September 2019 and June 2021.	A prospective, double-blinded randomized study	50	• The study included children diagnosed with posterior urethral valves (PUV) who were treated with endoscopic ablation using a cold knife. • These patients were followed clinically for voiding symptoms, with ultrasonography to assess residual urine and laboratory tests, particularly creatinine levels	*	Group A was given alpha-blockers. It was a randomized double-blinded controlled study where 50 closed envelopes 25 of them containing tamsulosin.	Group B was given placebo for 1month.25 containing a placebo which we choose to be paracetamol 150 mg syrup.	• Marked improvement of obstructive symptoms in Group A reaching about 90% (21 patients), whereas no mentioned improvement in Group B was noticed with no side effects of both medication the alpha-blocker and the placebo during its use	1 month
Bajpai et al. 2021 [17]	India, between January 1998 to May 2014.	A retrospective case–control	93	All patients of COPUM with radiological evidence of bladder neck hypertrophy and minimal follow-up of 7 years.	Patients with urethral strictures, neurogenic bladder, and incomplete records	Group I was treated with a selective α−1 blocker (prazosin), after valve ablation. Prazosin was started at a dose of 20 mcg/kg/day increasing up to 50 mcg/kg/day over 1 week.	Group II who had not received α−1 blocker was treated as a control group	• A total of 113 patients of COPUM were treated from January 1998 to May 2014. Out of these 113, 93 patients (82.3%) were included in the study. Fifty-seven (61.2%) received α−1 blocker, while 36 patients (38.8%) acted as control. • Significant decrease in bladder neck hypertrophy noted in Group I as compared to Group II (*P* < 0.001).	12 months
Abraham et al. 2009 [15]	India, between January 2002 and December 2007	A retrospective study	42	Children treated for posterior urethral valves (PUV) between January 2002 and December 2007 who were followed up regularly in the outpatient department.	*	• Children with significant postvoid residual urine were placed on Tablet Terazosin, a selective a1 adrenergic blocker dose ranging from 0.25 to 2 mg (0.02 to 0.4 mg/kg OD). • Tablet was powdered and mixed with water and appropriate dose was given. • Balance dissolved drug was discarded. • The Terazosin dose was adjusted according to PVR, as determined at the follow up clinic every 2 weeks. • Terazosin dose was increased in steps of 25% of the existing dose till the PVR became normal. • Parents were informed about the common side effects of hypotension after the initial dose and possible fainting attacks due to postural hypotension later in older boys so that they can report early if any. • Since hypotension is known to occur with the first dose of the drug, patients were kept under observation in the out patient department for 2 h after the first dose was given. • Once normal PVR is achieved and maintained for 6 months, tapering of the dose was attempted with monitoring of PVR. • Dose was progressively reduced and stopped if PVR remained normal. • This was possible because of the reduction in the bladder neck hyper trophy since outlet obstruction was corrected.	*	• Post void residual urine significantly reduced in 40 patients (95%) who were put on Terazosin. Mean pretreatment PVR was 15.7 ml and mean PVR at the last follow up was 2.4 ml (*P* = 0.000). • This was a reduction of 85% in the pretreatment post void residual urine volume. • All the patients had improvement in urinary stream. • One patient reacted to Terazosin with hypotension necessitating its withdrawal.34	Mean of 17 months
Singh et al. 2017 [18]	India, between January 2009 and December 2012	A prospective cohort study	71	• Children treated for posterior urethral valves with no residual valves confirmed by VCUG or check cystoscopy were included in the study. • Assessments were conducted two weeks after valve ablation, ensuring bladder neck and urethral spasm had subsided. • Post-void residual urine (PVR) was measured in toilet-trained children via uroflowmetry and in toilet-untrained children via ultrasound after a witnessed void.	• Children with residual posterior urethral valves not ruled out were excluded from the study. • Additionally, patients who did not adhere to regular follow-up schedules or experienced adverse events requiring the discontinuation of Tamsulosin were not included.	• Children less than 2 years of age and children more than 2 years of age, with significant post void residual urine were placed on 0.1 mg and 0.2 mg capsule Tamsulosin respectively. • Post void residual urine (PVR) and uroflowmetry was done at the commencement of alpha blocker and at follow up. • PVR was monitored with abdominal ultrasound by two different observers. • We also look for breakthrough urinary tract infection in children during the study.	*	• Mean pretreatment PVR was 31.59+/- 1.30 ml and mean PVR, at the last follow up was 7.62+/- 4.36 ml. (*P* = 0.000) There was a reduction of 75.87% in the pretreatment post void residual urine volume. • Mean pretreatment Qmax was 10.77 +/−6.57 ml/min and it was 13.78 +/−8.58 ml/min at the last follow up in toilet trained children. (*P* = 0.000) 34 patients had history of recurrent urinary tract infection. • At last follow up, there was resolution of breakthrough urinary tract infection.	Mean of 36 months
Bade et al. 2024 [16]	India	Randomized clinical trail	20	• Patients who underwent valve ablation, were toilet trained, had no end-stage renal disease, exhibited abnormal urodynamic study (UDS) findings (such as small bladder capacity, poor bladder compliance, high residual volume, or leakage during the study), and provided informed consent.	• Patients with residual valves, prior diversion procedures, end-stage renal disease, high-grade (grade IV/V) VUR, normal UDS (defined later), and larger-than-expected capacity bladder (described later) were excluded from the study.	• The group B patients were administered selective α−1 antagonist, terazosin, 0.2–0.4 mg/kg per day once daily. • Initially, terazosin was started at 0.02 mg/kg per dose and gradually increased over 2 weeks to a maximum dose of 2 mg once a day	*	• In group A, the mean maximum detrusor pressure (Pdet) decreased from 30.17 to 23.45 cm H2O (*p* = 0.033). • Two patients normalized from high detrusor pressure (> 40 cm H2O). In group B, 1 patient retained high detrusor pressure posttreatment. • Group B improved in average urinary flow (Q avg) and maximum flow rate (Q max), with all patients having initially low Q avg (< 10 mL/s). • Two group B patients showed improved average flow rates posttreatment (*p* = 0.016); three in group A showed improvement but were not statistically significant (*p* = 0.197). • Q max/flow time ratio was abnormal in all group B patients pretreatment. • Two of the nine improved posttreatment, while only one in group A did.	6 months
Mokhless et al. 2014 [6]	Egypt	Randomized clinical trail	20	Twenty children with severe bladder dysfunction and unfavorable clinical course after primary valve ablation were included	Any child with a history of urinary diversion (vesicostomy or ureterostomy) was excluded from the study	• The first group (study group) included 10 children with severe bladder dysfunction who underwent cystourethroscopy and simultaneous injection of 100 IU of Botox (Allergan, Irvine, CA, USA) into the hypertrophied bladder neck at 3, 6, 9 o’clock position and follow up.	All children had primary valve ablation at an age between 1 month and 24 months (mean 9 months)	• There was no statistical difference in both groups regarding rate of urinary tract infection, improvement of hydronephrosis, resolution of vesico-ureteral reflux, and creatinine level at the start or at the end of the study. • Urodynamic parameters revealed an increase in cystometric capacity in both groups at the end of the study but without statistical difference. • The mean voiding pressure reduced significantly in both groups but without statistical difference	6 months

The baseline data showed a broad age range, from 7.2 to 48.9 months, and serum creatinine levels between 0.59 and 1.24 mg/dL. Urodynamic findings highlighted variations in bladder function, including instances of elevated detrusor pressure, bladder hypercontractility, and significant residual volumes. Details of these findings are presented in Table 2.

**Table 2:** Baseline characteristics of included studies

Study ID		Number of the patients	Age (months) mean&SD	Baseline serum creatinine (mg/dL) mean&SD	Baseline VUR (renal units) (*n* (%))	Maximum detrusor pressure at Qmax (Pdetmax) (cm H2O) mean&SD	No of Signifcant Residual volume (*n* (%))	Detrusor overactivity (*n* (%))	Preoperative PVR (ml) mean&SD	Qmax (ml/s) mean&SD	No. Myogenic Failure (*n* (%))	Baseline Hydronephrosis (*n* (%))
Abdelhalim et al. 2022 [7]	Bladder neck incision with valve ablation	44	7.85 ± 5.72	0.59 ± 0.33	24 (54.55)^	*	*	*	*	*	*	*
	Valve ablation only	70	8.64 ± 4.85	0.59 ± 0.44	28 (40)	*	*	*	*	*	*	*
Singh et al. 2019 [9]	Bladder neck incision with valve ablation	34	29.4	0.9	24 (70.59)	202 ± 32	*	*	*	*	*	*
	Valve ablation only	37	30.6	0.92	21 (56.76)	194 ± 39	*	*	*	*	*	*
Sarin et al. 2013 [11]	Bladder neck incision with valve ablation	9	48.98 ± 39.82	*	5 (27.78)	*	*	*	*	*	*	15 (83.33)
	Valve ablation only	9	44.66 ± 20.48	*	0	*	*	*	*	*	*	9 (50)
Kajbafzadeh et al. 2007 [10]	Bladder neck incision with valve ablation	22	18.84 ± 18.75	*	17 (38.64)	235 ± 41	1 (4.55)	11 (50)	*	*	0	18 (81.82)
	Valve ablation only	24	15.71 ± 14.64	*	18 (37.5)	231 ± 51	1 (4.17)	11 (45.83)	*	*	0	20 (83.33)
Sobhan et al. 2024 [13]	Bladder neck incision with valve ablation	301	7.22 ± 2.45	*	188 (62.46)	288.66 ± 42.31	15 (4.98)	189 (62.79)	*	*	16 (5.32)	266 (88.37)
Aboulela et al. 2024 [14]	Alpha blocker group	25	8.3 ± 12.6	*						*		
	Valve ablation only	25		*	*	*				*		*
Bajpai et al. 2021 [17]	Alpha blocker group	57	42.72 ± 34.08	*	21 (22.58)	*			110 ± 22.62	8.5 ± 2.71		*
	Valve ablation only	36	45.72 ± 12.24	*		*			116 ± 11.24	7.1 ± 1.9		*
Abraham et al. 2009 [15]	Alpha blocker group	42	15 ± 26.88	*	*	*	*	*	15.7	*	*	*
Singh et al. 2017 [18]	Alpha blocker group	71	89.4 ± 46.6	*	*	*	*	*	31.59 ± 1.3	10.77 ± 6.57	*	*
Bade et al. 2024 [16]	Alpha blocker group	9	4.1 ± 9.74	0.56 ± 0.12	*	33.44 ± 5.05	*	*	*	11.77 ± 4.08		*
Mokhless et al. 2014 [6]	Botox group	10	16 ± 9.74	0.87 ± 0.34	8 (40)	*	*	8 (80)	*	*	*	*
	Valve ablation only	10		1.24 ± 0.42	10 (50)	*	*	6 (60)	*	*	*	*

^ indicates the number of patients not in the renal unit

### Results of the Quality Assessment

**Fig. 2:**
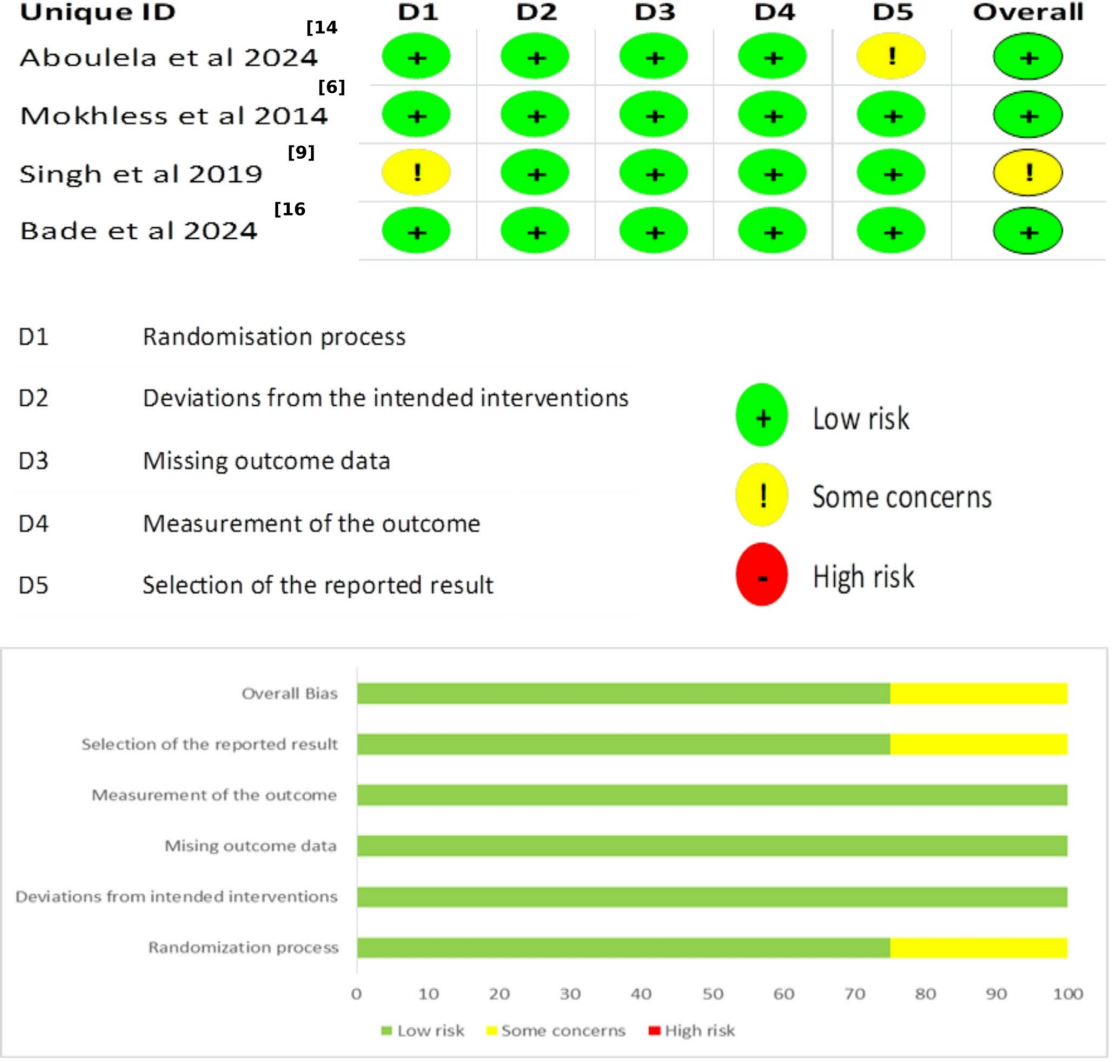
Quality Assessment Using Cochrane Risk of Bias 2 (ROB 2) Tool

Three studies were considered to have a low risk of bias based on the ROB2 tool [6, 13, 15, 27]. However, one study showed some concerns because it did not provide enough details on how randomization was handled, especially regarding the randomization of assessors, caregivers, and those providing the intervention [9] (Fig. 2).

The observational study, along with the pilot study, were both assessed as having good quality using the NIH [7, 10, 11, 16, 28] (Table 3).

**Table 3:** NIH quality assessment tool for the included studies

Study ID	Item 1	Item 2	Item 3	Item 4	Item 5	Item 6	Item 7	Item 8	Item 9	Item 10	Item 11	Item 12	Item 13	Item 14	Score
Abdelhalim et al. 2022 [7]	Yes	Yes	yes	yes	NR	yes	yes	Yes	yes	Yes	yes	NR	Yes	Yes	Good
Sarin et al. 2013 [11]	Yes	Yes	Yes	Yes	NR	yes	yes	yes	yes	yes	yes	NR	Yes	NR	Good
Kajbafzadeh et al. 2007 [10]	Yes	Yes	Yes	Yes	NR	yes	yes	yes	yes	yes	yes	NR	Yes	NR	Good
Bajpai et al. 2021 [17]	Yes	Yes	Yes	Yes	NR	yes	yes	yes	yes	yes	yes	NR	Yes	NR	Good

*CD* cannot determine, *NA* not applicable, *NR* not reported

Item 1: Research question;

Item 2 and 3: Study population;

Item 4: Groups recruited from the same population and uniform eligibility criteria;

Item 5: Sample size justification;

Item 6: Exposure assessed prior to outcome measurement;

Item 7: Sufficient timeframe to see an effect;

Item 8: Different levels of the exposure of interest;

Item 9: Exposure measures and assessment;

Item 10: Repeated exposure assessment;

Item 11: Outcome measures;

Item 12: Blinding of outcome assessors;

Item 13: Follow-up rate;

Item 14: Statistical analyses.

## Outcomes

### Concomitant BNI with VA

#### VUR Resolution (Number of renal units with resolved vesicoureteral reflux (VUR)

##### Double-Arm Analysis

**Fig. 3:**
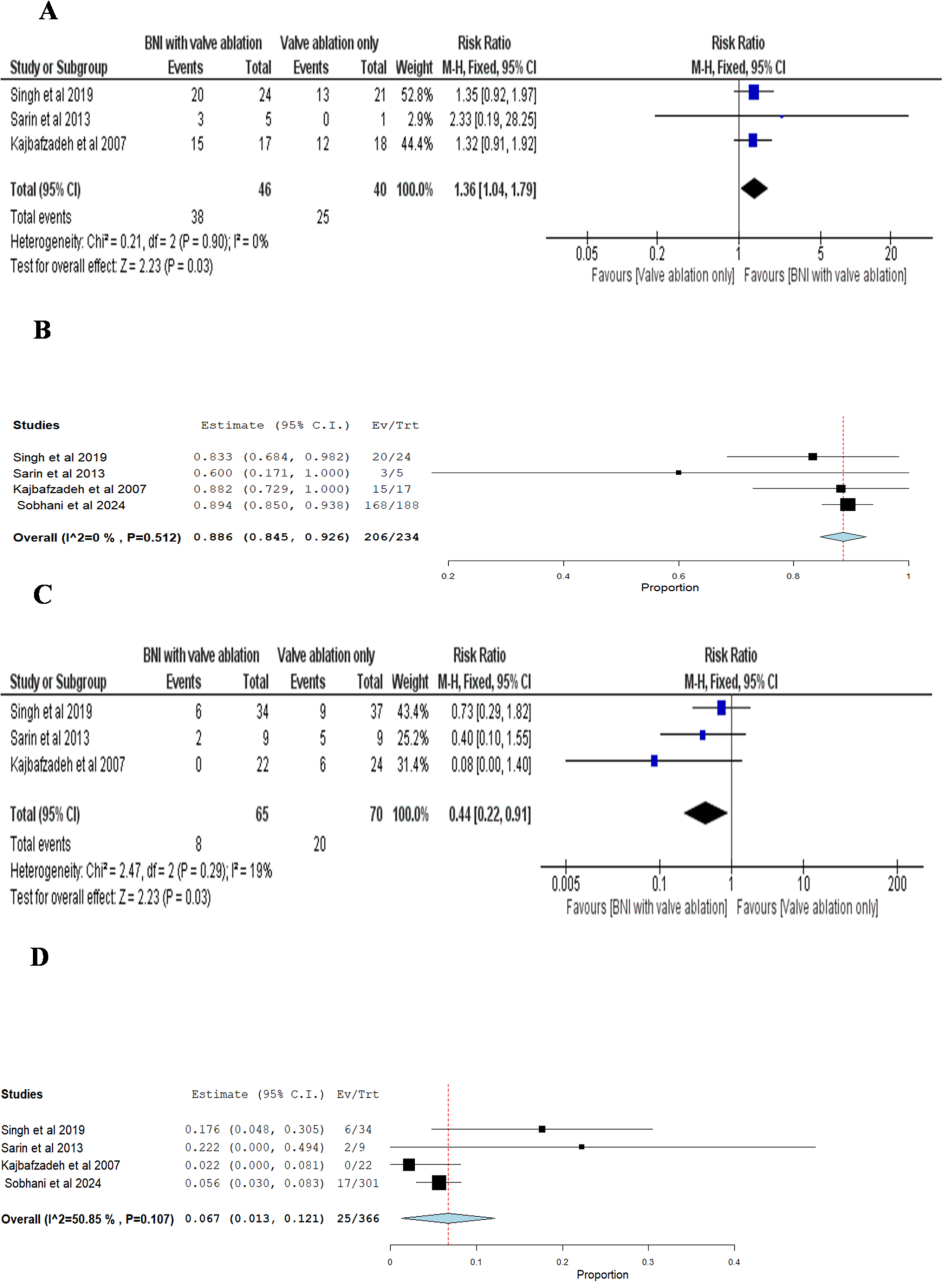
(**A**) Double-arm meta-analysis of vesicoureteral reflux (VUR) resolution, comparing bladder neck incision (BNI) combined with valve ablation versus valve ablation alone. Events refer to the number of renal units in which VUR resolved postoperatively (**B**) Single-arm meta-analysis of VUR resolution in renal units following BNI combined with valve ablation. Events represent the number of renal units with resolved VUR after treatment (**C**) Double-arm meta-analysis of postoperative detrusor overactivity (DOA) comparing BNI with valve ablation versus valve ablation alone. Events refer to the number of patients demonstrating postoperative DOA (**D**) Single-arm meta-analysis of postoperative DOA in patients undergoing BNI with valve ablation. Events refer to the number of patients demonstrating postoperative DOA

 The pooled Risk Ratio (RR) of three studies favored BNI combined with valve ablation over valve ablation only group, indicating a significantly higher rate of VUR resolution compared to valve ablation alone (RR = 1.36; 95% CI: 1.04 to 1.79; p = 0.03). The studies were homogeneous, with an I² of 0% and a p-value for heterogeneity of 0.90 Fig. 3A.

##### Single-Arm Analysis

 The pooled estimate of four studies for BNI combined with valve ablation was 0.886 (88.6%) (95% CI: 0.845 to 0.926), with no substantial heterogeneity observed (I² = 0%, p =0.512) Fig. 3B.

####  Postoperative Detrusor Overactivity (DOA) (Number of patients demonstrating postoperative DOA)

##### Double-Arm Analysis

The pooled Risk Ratio of three studies favored BNI combined with valve ablation over valve ablation only group, indicating that this group had significantly less DOA compared to the valve ablation only group (RR = 0.44; 95% CI: 0.22 to 0.91; p = 0.03). The studies were homogeneous, with an I² of 19% and a p-value for heterogeneity of 0.29 Fig. 3C.

##### Single-Arm Analysis

The pooled estimate of four studies for BNI combined with valve ablation was 0.067 (6.7%) (95% CI: 0.013 to 0.121), with no significant heterogeneity observed (I² = 50.85%, p =0.107) Fig 3D.

#### Number of patients who required re-intervention

##### Double-Arm Analysis

**Fig. 4:**
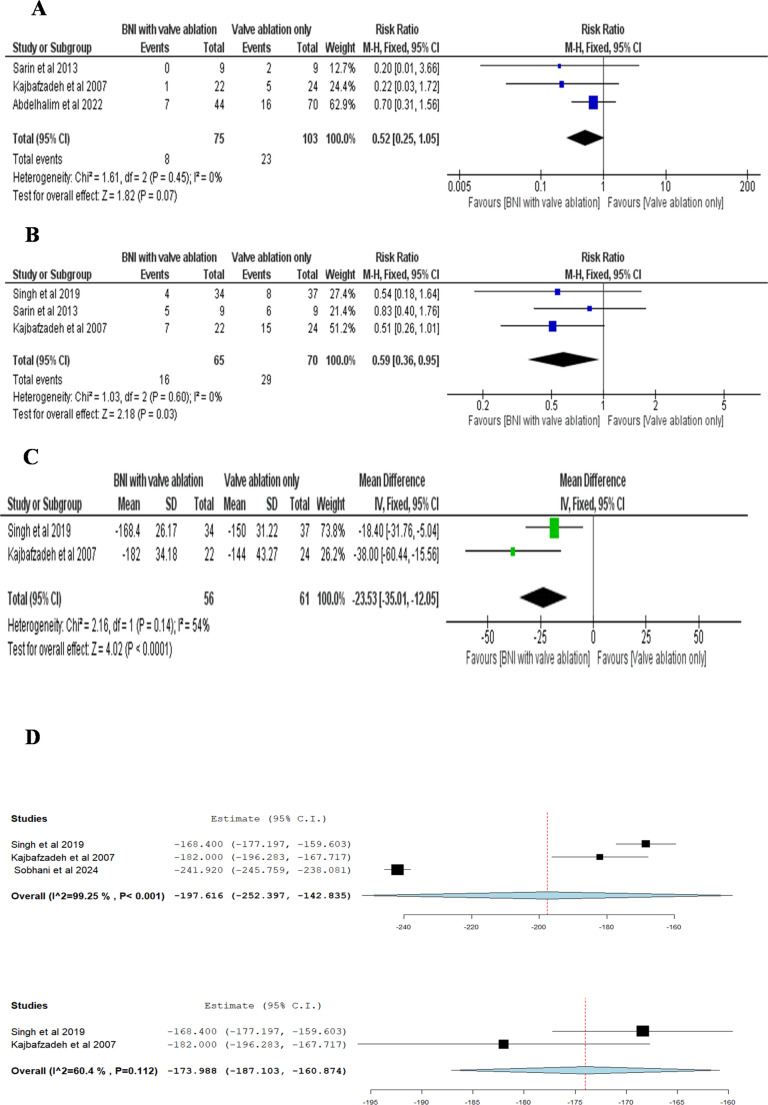
(**A**) Double-arm meta-analysis of number of patients who required re-intervention, comparing bladder neck incision (BNI) combined with valve ablation versus valve ablation alone. Events refer to the number of patients who needed any additional surgical or medical intervention after initial treatment (**B**) Double-arm meta-analysis of number of patients requiring alpha-blockers or anticholinergics after treatment. Events represent patients who needed these medications postoperatively, comparing BNI plus valve ablation to valve ablation alone (**C**) Double-arm meta-analysis of change in maximum detrusor pressure at Qmax (Pdetmax), comparing BNI combined with valve ablation versus valve ablation alone. This is reported as the mean difference in pressure reduction (**D**) Single-arm meta-analysis of change in Pdetmax following BNI combined with valve ablation. The pooled mean difference in detrusor pressure reduction is shown with heterogeneity estimates

The pooled Risk Ratio (RR) from three studies indicated no statistically significant difference in the rate of re-intervention between patients who underwent BNI combined with valve ablation and those who received valve ablation alone. (RR = 0.52; 95% CI: 0.25 to 1.05; p = 0.07). The studies were homogeneous, with an I² value of 0% and a p-value for heterogeneity of 0.45 Fig. 4A.

#### Number of patients who required alpha blocker/anticholinergic

##### Double-Arm Analysis

The pooled Risk Ratio of three studies favored BNI combined with valve ablation over valve ablation only, indicating that patients in the BNI group were significantly less likely to require alpha blocker or anticholinergic therapy. (RR = 0.59; 95% CI: 0.36 to 0.95; p = 0.03). The studies were homogeneous, with an I² of 0% and a p-value for heterogeneity of 0.60 Fig. 4B.

#### Change in P detmax

##### Double-Arm Analysis

The pooled mean difference of two studies favored BNI combined with valve ablation over valve ablation alone, with a significant reduction of**−23.53 cm H₂O** (95% CI: **−35.01** to **−12.05**;**p< 0.0001**). No significant heterogeneity was detected, as indicated by an **I²** value of **54%** and **a p-value** of **0.14** Fig. 4C.

##### Single-Arm Analysis

The pooled estimate of three studies for BNI combined with valve ablation was **−197.616cm H₂O** (95% CI: −252.397 to −142.835), with significant heterogeneity observed (I² = 99.25%, p < 0.001). After excluding Sobhani et al. in the leave-one-out test, the pooled estimate was **−173.988** (95% CI: −187.103 to −160.874), with reduced heterogeneity (**I²= 60.4%, p = 0.112**) Fig. 4D.

#### Postoperative serum creatinine

##### Double-Arm Analysis

**Fig. 5:**
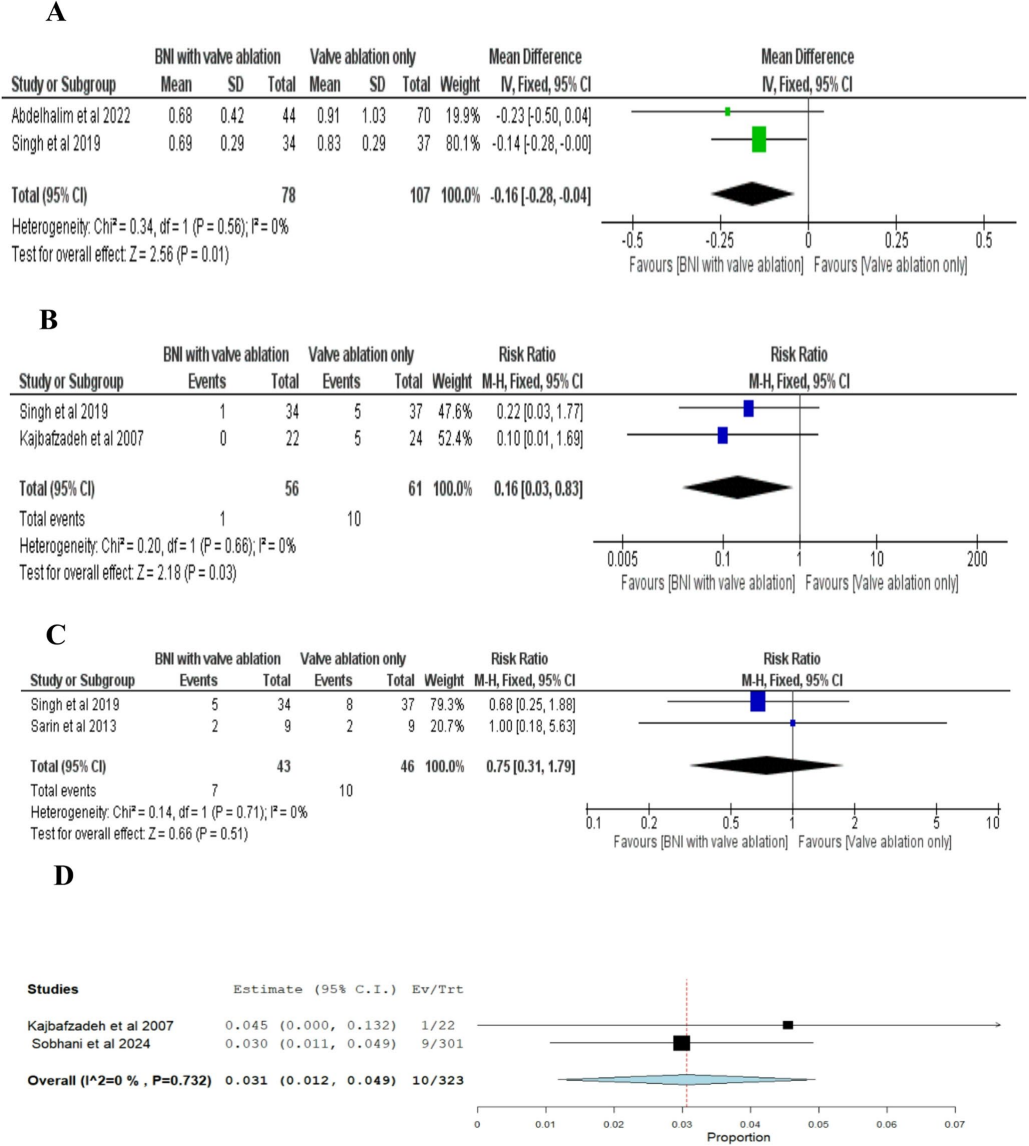
**A**) Double-arm analysis of postoperative serum creatinine levels in patients undergoing BNI combined with valve ablation versus valve ablation alone **B**) Double-arm analysis of the number of patients requiring clean intermittent catheterization (CIC) postoperatively. Events refer to individual patients who required CIC during follow-up ** C**) Double-arm analysis of the number of patients with poor bladder compliance postoperatively **D**) Single-arm analysis of postoperative post-void residual (PVR) volume in patients undergoing BNI combined with valve ablation. Values are reported as proportions of patients with elevated PVR

The pooled mean difference of two studies favored BNI combined with valve ablation over valve ablation alone, with a significant reduction of−0.16 mg/dL (95% CI: −0.28 to −0.04; p =0.01). No significant heterogeneity was detected, as indicated by an I² value of 0% and a p-value of 0.56 Fig. 5A.

#### Number of patients who required CIC

##### Double-Arm Analysis

The pooled Risk Ratio of three studies showed that BNI combined with valve ablation significantly reduced the need for clean intermittent catheterization (CIC) compared to valve ablation alone (RR = 0.16; 95% CI: 0.03 to 0.83; p = 0.03). The studies were homogeneous, with an I² of 0% and a p-value for heterogeneity of 0.66 Fig. 5B.

#### Poor bladder compliance

##### Double-Arm Analysis**(Number of patients with poor bladder compliance postoperatively)**

The pooled Risk Ratio (RR) from three studies indicated no significant difference between BNI combined with valve ablation and valve ablation alone (RR = 0.75; 95% CI: 0.31 to 1.79; p = 0.051). Across these studies, 7 out of 43 patients (16.3%) in the BNI group had poor bladder compliance, compared to 10 out of 46 patients (21.7%) in the valve ablation only group. The studies were homogeneous, with an I² value of 0% and a p-value for heterogeneity of 0.71 Fig. 5C.

#### Postoperative PVR

##### Single-Arm Analysis (number of patients with significant PVR)

The pooled estimate of two studies for BNI combined with valve ablation was 0.031 (3.1%) (95% CI: 0.012 to 0.049), with no substantial heterogeneity observed (I² = 0%, p =0.732) Fig. 5D.

 Singh et al. reported that the BNI combined with valve ablation group had a significantly lower mean post-void residual (PVR) volume of 15.41 ml, compared to 21.15 ml in the valve ablation only group (p = 0.045)

#### Myogenic failure (Number of patients demonstrating myogenic failure postoperatively)

##### Single-Arm Analysis

**Fig. 6:**
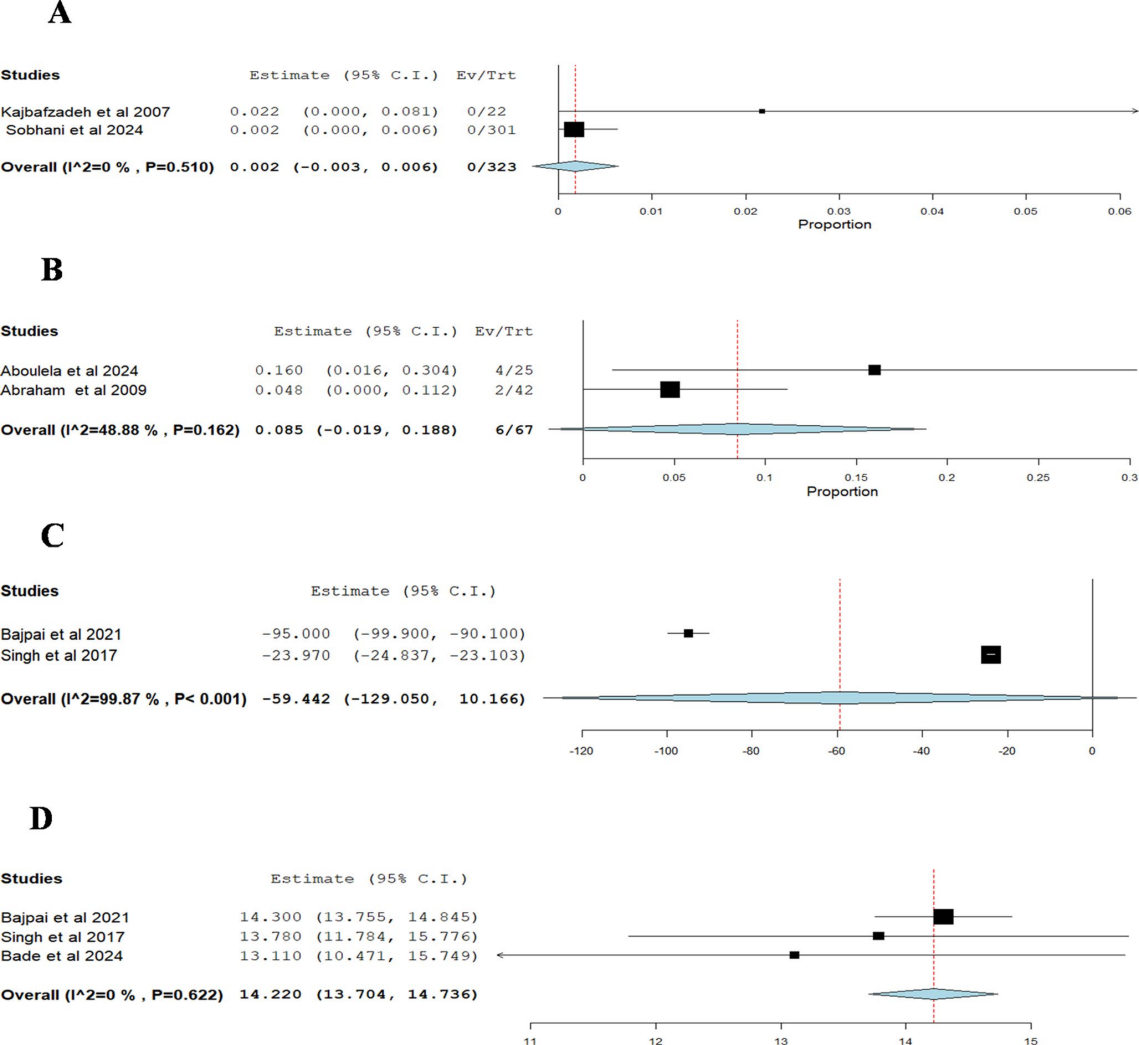
(**A**) Single -arm analysis of Myogenic failure for BNI combined with valve ablation, Events refer to the number of patients demonstrating myogenic failure postoperatively. (**B**) Single -arm analysis of Postoperative PVR, *Events* refer to the number of patients with significant PVR for Alpha blockers therapy after valve ablation, (**C**) Single-arm analysis of Change in PVR for Alpha blockers therapy after valve ablation, (**D**) Single-arm analysis of Postoperative Qmax for Alpha blockers therapy after valve ablation

The pooled estimate of two studies for BNI combined with valve ablation was 0.002 (0.2%) (95% CI: −0.003 to 0.006), with no substantial heterogeneity observed (I² = 0%, p =0.510) Fig. 6A.

#### Peak flow rate (Qmax)

 Singh et al. was the only study to report the Qmax, with the BNI combined with valve ablation group showing a significantly higher Qmax (10.92 ml/min) compared to the valve ablation only group (6.91 ml/min, p = 0.038). The mean age at the time of surgery was 2.45 years in the BNI group and 2.55 years in the valve ablation-only group. At the end of follow-up, the mean age was 7.26 years in the BNI group and 7.66 years in the valve ablation-only group.

### Alpha Blockers Therapy After Valve Ablation

#### Postoperative PVR

##### Single-Arm Analysis (number of patients with significant PVR):

The pooled estimate of two studies for Alpha blockers intervention after valve ablation was 0.085 (8.5%) (95% CI: −0.019 to 0.188), with no significant heterogeneity observed (I² = 48.88%, p =0.162) Fig. 6B.

#### Change in PVR

##### Single-Arm Analysis

The pooled estimate of two studies for Alpha blockers intervention after valve ablation was −59.442 ml (95% CI: −129.050 to 10.166), with significant heterogeneity observed (I² = 99.87%, p >0.001) Fig. 6C.

 Bajpai et al. reported that the alpha blocker therapy group showed a significant reduction in post-void residual (PVR) volume from 110±22.62 ml to 15±8.21 ml, compared to the valve ablation only group, which had a decrease from 116±11.24 ml to 54±13.87 ml (P < 0.01).

 Abraham et al. reported in a single-arm study that the mean post-treatment post-void residual (PVR) was 2.4 ml, significantly lower than the pre-treatment PVR of 15.7 ml (P = 0.000).

#### Peak flow rate (Qmax)

##### Single-Arm Analysis

The pooled estimate of three studies for alpha blockers intervention after valve ablation was 14.220 ml/sec (95% CI: 13.704 to 14.736), with no substantial heterogeneity observed (I² = 0%, p =0.622) Fig.6D.

 Bade et al. reported that in the alpha-blocker intervention after valve ablation group, the mean Qmax increased from 11.77 mL/s pre-therapy to 13.11 mL/s post-intervention, with 66.6% of patients achieving Qmax >10 mL/s. The change in Qmax was statistically insignificant (**p= 0.07**).

Bajpai et al. reported that in the alpha-blocker intervention after valve ablation group, the peak urine flow rate increased from 8.50±2.71 mL/s post-valve ablation to 14.3±2.1 mL/s at the last follow-up. In the valve ablation only, the peak urine flow rate increased from 7.1±1.9 mL/s post-ablation to 8.7±2.3 mL/s at the last follow-up. The difference between the intervention and control groups was not statistically significant (**p>0.05**).

#### Change in P detmax

 Bade et al. reported that in the alpha-blocker intervention group after valve ablation, the mean Pdetmax was 33.44 ±5.05 cm H₂O and reduced to 23.77±10.74 cm H₂O The fall in mean Pdetmax was 28.9% and was statistically significant (**p= 0.028**).

### Botox injection at the bladder neck

 Mokhless et al. was the only included study that assessed Botox injection at the bladder neck and reported the following outcomes, with urodynamic studies performed at baseline and reassessed at 6 months:

i. Among the 20 renal units in the Botox injection after valve ablation group, 8 units (40%) exhibited vesico-ureteral reflux, compared to 10 out of 20 units (50%) in the valve ablation only group. These numbers remained consistent by the end of the study, with high-grade reflux (grades 4 and 5) observed in both groups.
ii. Detrusor overactivity decreased to 3 cases in the Botox injection after valve ablation group and resolved completely in the valve ablation only group. The difference between the groups was not statistically significant (p = 0.211).
iii. The mean voiding pressure was 70.7 ± 23.79 cmH2O in the Botox injection after valve ablation group and 65.4 ± 14.14 cmH2O in the valve ablation only group. The difference between the groups was not statistically significant (p = 0.074)
iv. Post-voiding residual volume (PVR) was significant in 7 cases (70%) of the Botox injection after valve ablation group compared to 5 cases (50%) in the valve ablation only group. The mean PVR was 55.56 ± 34.68 for the Botox injection after valve ablation group and 40.50 ± 24.43 for the valve ablation only group. The difference in PVR between the groups was not statistically significant (p = 0.565).
v. Poor compliance was observed in 1 case in the Botox injection after valve ablation group and 1 case in the valve ablation only group. The difference between the groups was not statistically significant (p = 1.000)

## Discussion

VBS defined as pathophysiological bladder changes with persistent hydroureteronephrosis and renal impairment could occur even after valve ablation as a result of renal tubular dysfunction and polyuria, high bladder filling pressures secondary to a non-compliant bladder, and poor bladder emptying due to outlet obstruction secondary to residual valve tissue, urethral stricture or bladder neck obstruction [1, 4]. This is a dynamic process that may change over time and perhaps not even appear till later in life.

Secondary BNO in VBS is an urodynamic based, not endoscopically nor radiographically based diagnosis. BNO can present with a normal Qmax if a strong contraction overcomes the obstruction, but Qave is typically abnormal. Voiding pressure and uroflow rates (Qmax/Qave), ideally with videourodynamics, are crucial for detecting obstruction [1, 5].

Significant hypertrophy semi-closed bladder necks in some patients with PUV and myogenic failure has been discovered by Androulakakis et al. Voiding in these conditions may lead to obstruction at the bladder neck, resulting in detrusor dysfunction [8]. While some studies have shown that BNI performed during childhood does not adversely affect retrograde ejaculation or urinary continence in adulthood, there are concerns regarding the risk of incontinence and retrograde ejaculation when BNI is performed concurrently with VA in children with PUV [4, 7]. Misseri et al. applied BNI in a patient with secondary bladder neck obstruction, and Androulakakis et al. applied BNI for a patient with myogenic failure after VA, and both had improvements in bladder function [8]. Concomitant BNI and VA may improve urodynamic outcomes and reduce both the risk of bladder dysfunction and re-intervention in PUV patients [9, 10].

On the other hand Sarin and Sinha found no significant difference between concomitant BNI and VA and VA only regarding the urodynamic outcomes [11]. Notably, evidence indicates that concomitant BNI and VA do not reduce early hospital readmission or re-intervention rates compared to VA alone [7].

Bladder neck incision (BNI) could disrupt the vesicourethral function, a system that typically maintains continence even when the urethral sphincters are accidentally or intentionally damaged. It also has a role in the sperm propulsion [29].

Long-term complications have been reported in the broader literature, including incontinence and retrograde ejaculation [10, 30–32]. Bladder neck contraction and coordinated contraction of the structures around the verumontanum serve the principal role in antegrade semen movement [33]. After bilateral BNI in adult men, retrograde ejaculation has been reported in 23–27%, while Moisey et al. described a 16% incidence following unilateral BNI [32, 34, 35]. Unilateral BNI in adults has also been associated with reduced sperm count, to about 69% at follow-up [36].In contrast, Keihani et al. reported reassuring long-term functional outcomes in a small series of adult patients who had undergone BNI at the 6 o’clock position proximal to the verumontanum, with no cases of incontinence or dry ejaculation and only one patient reporting weak ejaculation [33]. Similarly, Hennus et al. evaluated men nearly 20 years after undergoing unilateral superficial BNI in childhood and found that all maintained antegrade ejaculation, with only 10.8% reporting reduced ejaculatory volume, 5.4% reporting moderate incontinence, and 22% experiencing moderate lower urinary tract symptoms [37].

To protect ejaculation and continence, some pediatric urologists recommend alpha blockers to address secondary bladder neck obstruction. Combs reported improved voiding pressures and flow rates with this approach [38]. Mokhless et al. evaluated bladder neck botulinum toxin injections in 10 patients with dysfunction post-VA and reported no impact on urodynamics, hydronephrosis, or reflux resolution [6].

Secondary bladder neck obstruction defined as increased voiding Pdet and obstructed uroflow in the absence of residual PUV and urethral stricture.” Secondary BNO is a urodynamic diagnosis, ideally with video urodynamics, and not a diagnosis that can be made endoscopically or radiographically. Improved peak flow and PVR are not sure but can aid the diagnosis of BNO [1, 4, 5].

Concomitant BNI intervention and VA decreased bladder hypercontractility, voiding pressure and myogenic failure [39]. However, a randomized controlled trial showed improvements only in peak flow and post-void residual without improvements in other urodynamic variables [9]. In a randomized controlled trial, Botox injection at BN after VA did not significantly alter detrusor overactivity, mean voiding pressure, or bladder compliance compared to VA only [6]. This meta-analysis showed that concomitant BNI intervention and VA significantly decreased detrusor overactivity and maximum detrusor storage pressure (Pdetmax), without any significant effect on bladder compliance, compared to VA only; these outcomes were assessed at follow-up durations ranging from 3 months to approximately 60 months across the included studies [9–11].

Despite high voiding pressures and small bladder capacities at diagnosis, 75% of PUV patients show elevated PVR. Three years after diagnosis and VA, half of the patients with age-appropriate bladder capacity still exhibit high PVR [40, 41]. This meta-analysis found that both BNI and alpha blocker significantly decrease the PVR volume. Botox injection at BN did not significantly alter the PVR volume after VA. The mean PVR was 55.56 ± 34.68 for the Botox injection after VA and 40.50 ± 24.43 for VA only group [6].

Secondary VUR occurred in one third of PUV cases and is linked to poor kidney function, particularly in bilateral VUR. Spontaneous resolution of VUR occurs in 27% to 79% of cases within 2 weeks to over a year after VA. Resolution was significantly faster for unilateral VUR, and for low grades, and was not influenced by ipsilateral renal function [42]. VUR resolution was evaluated by VCUG at baseline and follow up to evaluate the VUR grade. VUR resolution was evaluated after 3 months in Sarin et al. trial [11]. VUR resolution was evaluated at 6 weeks in Singh et al. study [9]. In Kajbafzadeh study, VUR resolution was evaluated at 3rd,6th, and 8th month if VUR persistence was present [10].

Botox injection at BN after VA did not change the number or the degree of VUR at 6th month of follow up [6]. In our meta-analysis concomitant BNI with VA showed a statistically significant difference versus VA only, regarding the resolution of VUR. This better improvement might attributed to that with concomitant BNI with VA the urethral obstruction has been completely eliminated with ablation of the obstructed valve and incision of the bladder neck which might enhance the reflux resolution.

PUV late presentation, VA after one month, high initial serum creatinine, persistence of high serum creatinine after one-month post-VA are poor renal outcomes predictors with other comorbidities as VUR, urosepsis and renal dysplasia. In obstructive nephropathy of PUV tubulopathy precedes glomerulopathy, also renal injury continues even after VA [43].

Nadir creatinine within six weeks of VA could predict CKD severity [44]. Children with a post-VA nadir creatinine above 0.85 mg/dL were at increased risk for CKD [45]. Botox injection at BN after VA had no statistical difference regarding serum creatinine versus VA only. In this systematic review and meta-analysis, concomitant BNI with VA showed a statistically significant reduction in serum creatinine versus VA only (*p* = 0.01).

Obstructions in the sphincter area and proximal urethra may have normal or near normal Qmax caused by high detrusor pressures. Maximum flow rate (Qmax) alone can diagnose only 81.0% of PUV [46]. In this systematic review and meta-analysis, the pooled estimate of post-treatment maximum urinary flow rate (Qmax) from three studies evaluating alpha-blocker therapy after valve ablation was 14.220 mL/Sect. (95% CI: 13.704 to 14.736).

This systematic review has a number of strengths. Firstly, the paper adheres with the current reporting guidelines for systematic reviews and meta-analyses. It is the first meta-analysis to comprehensively evaluate bladder neck interventions. Furthermore, it includes all relevant studies on these interventions, ensuring a thorough and robust synthesis of the available evidence. We reported a range of outcomes. Ultimately, our work aims to fill a critical gap in the existing body of research.

However, we faced some limitations, including the absence of randomized controlled trials (RCTs) on this approach. We were only able to include four RCTs, one of which had some concerns regarding its methodology. We also had to include available retrospective cohort studies, and our analysis was constrained by a limited sample size. Additionally, one study grouped alpha blockers and anticholinergics together, which limited our ability to analyze them separately—despite their different clinical roles [9]. The timing of outcome assessment (e.g., short-term vs. longer-term follow-up) varied across studies, and, given the relatively small number of included studies, we were unable to conduct subgroup analyses by follow-up duration. This should be taken into account when considering the pooled estimates.Well-structured randomized controlled trials with larger participant groups and longer follow-up periods are needed to better assess outcomes such as urinary incontinence and retrograde ejaculation following BNI combined with VA.

## Conclusion

In the included studies, BNI combined with VA could significantly improve bladder function regarding VUR resolution, detrusor overactivity, and maximum detrusor pressure at Qmax. It also could significantly decrease both the use of alpha blocker/anticholinergic, and the need for intermittent catheterization. However, there was no difference either in bladder compliance, nor re-intervention rate. While improvements in certain urodynamic parameters were evident, incontinence outcomes were not reported in the included studies and thus could not be evaluated.

## Key References

- Chan EP, Wang PZT, Dave S Valve bladder syndrome associated with posterior urethral valves: natural history, work-up, and management. Current Bladder Dysfunction Reports 15:76-82.2020
  - ○ This is a narrative review focus on diagnosis, evaluation, and management of valve bladder syndrome following posterior urethral valves ablation.
- Abdelhalim A, Hashem A, Abouelenein EE, Atwa AM, Soltan M, Hafez AT, Dawaba MS, Helmy TE Can Concomitant Bladder Neck Incision and Primary Valve Ablation Reduce Early Re-admission Rate and Secondary Intervention? Int Braz J Urol 48 (3):485-492.2022
  - ○ This a retrospective study have shown that there is no need to perform BNI together with PUV ablation as patient perform concomitant BNI and valva ablatin had the same reoperation rate as those who underwent PUV alone.
- Holmdahl G, Sillen U, Bachelard M, Hansson E, Hermansson G, Hjalmas K, Bauer SB The changing urodynamic pattern in valve bladders during infancy. The Journal of urology 153 (2):463-467.1995
  - ○ This is a narrative review focus on using novel biomarkers of obstructive nephropathy to predict the onset and progression of chronic kidney disease in posterior urethral valves patients.

## Supplementary Information

Below is the link to the electronic supplementary material.

Supplementary Material 1 (DOCX 22.9 KB)

## Data Availability

The datasets used and/or analyzed during this study are available from the corresponding author upon reasonable request.
