# Supplementary material for: Bladder Neck Incision in Posterior Urethral Valve Management: a Meta-Analysis with Insights into Adjunctive Bladder Interventions
Source: Curr Urol Rep. 2025 Dec 23;27(1):1. doi: 10.1007/s11934-025-01309-w (PMC12727795; doi:10.1007/s11934-025-01309-w)
Supplement: Supplementary file 1 — Supplementary Material 1 (DOCX 22.9 KB) [file 11934_2025_1309_MOESM1_ESM.docx]

***Search strategy*:**

***PUBMED:***

(“Posterior urethral valves” OR “Posterior urethral valve” OR “PUVs” OR “PUV” OR “Congenital obstructing posterior urethral membrane” OR “COPUM” OR "Urethral Obstruction"[Mesh] OR "Urethral Diseases"[Mesh]) AND (“Valve Ablation” OR “urethral valve ablation” OR “primary valve ablation” OR “posterior urethral valve ablation” OR “Valve Resection” OR “Endoscopic Valve Incision” OR “Transurethral Valve Ablation” OR “Primary Valve Incision” OR “Endoscopic Ablation ” OR “Valve Fulguration” OR "Ablation Techniques"[Mesh]) AND (“Bladder Neck Incision” OR “BNI” OR “B.N.I.” OR “bladder-neck incision” OR “Bladder Neck Incisions” OR “Transurethral Incision” OR “Incisional Techniques” OR “Posterior Urethral Valve Incisions” OR “Alpha adrenergic blocker” OR “Alpha-blockers” OR “Alpha-1 blockers” OR “Alpha-1 adrenergic blockers” OR “α-1 Adrenergic Blockers” OR "Adrenergic alpha-Antagonists"[Mesh] OR “Anticholinergics” OR “Anticholinergic” OR “Oxybutynin” OR “Oxybutinin” OR “Botulinum A” OR “Onabotulinumtoxin A” OR “Botulinum toxin” OR “Botulinum Toxin A” OR “Botulinum Neurotoxin” OR “BoNTA” OR “Botox injection”)

*Date of search: November 2024*

*Search results: 82*

*No limitations applied*

***Cochrane library***

#1 (“Posterior urethral valves” OR “Posterior urethral valve” OR “PUVs” OR “PUV” OR “Congenital obstructing posterior urethral membrane” OR “COPUM”)

#2 (“Valve Ablation” OR “Urethral valve ablation” OR “Primary valve ablation” OR “Posterior urethral valve ablation” OR “Valve Resection” OR “Endoscopic Valve Incision” OR “Transurethral Valve Ablation” OR “Primary Valve Incision” OR “Endoscopic Ablation ” OR “Valve Fulguration”)

#3 (“Bladder Neck Incision” OR “BNI” OR “B.N.I.” OR “Bladder-neck incision” OR “Bladder Neck Incisions” OR “Transurethral Incision” OR “Incisional Techniques” OR “Posterior Urethral Valve Incisions” OR “Alpha adrenergic blocker” OR “Alpha-blockers” OR “Alpha-1 blockers” OR “Alpha-1 adrenergic blockers” OR “α-1 Adrenergic Blockers” OR “Anticholinergics” OR “Anticholinergic” OR “Oxybutynin “ OR “Oxybutinin” OR “Botulinum A” OR “Onabotulinumtoxin A” OR “Botulinum toxin” OR “Botulinum Toxin A” OR “Botulinum Neurotoxin” OR “BoNTA” OR “Botox injection”)

*#4 (#1 AND #2 AND #3)*

“Botox injection”)

*Date of search: November 2024*

*Search results: 3*

*No limitations applied*

***SCOPUS***

ALL ( ( "Posterior urethral valves" OR "Posterior urethral valve" OR "PUVs" OR "PUV" OR "Congenital obstructing posterior urethral membrane" OR "COPUM" ) AND ( "Valve Ablation" OR "Urethral valve ablation" OR "Primary valve ablation" OR "Posterior urethral valve ablation" OR "Valve Resection" OR "Endoscopic Valve Incision" OR "Transurethral Valve Ablation" OR "Primary Valve Incision" OR "Endoscopic Ablation " OR "Valve Fulguration" ) AND ( "Bladder Neck Incision" OR "BNI" OR "B.N.I." OR "Bladder-neck incision" OR "Bladder Neck Incisions" OR "Transurethral Incision" OR "Incisional Techniques" OR "Posterior Urethral Valve Incisions" OR "Alpha adrenergic blocker" OR "Alpha-blockers" OR "Alpha-1 blockers" OR "Alpha-1 adrenergic blockers" OR "α-1 Adrenergic Blockers" OR "Anticholinergics" OR "Anticholinergic" OR "Oxybutynin " OR "Oxybutinin" OR "Botulinum A" OR "Onabotulinumtoxin A" OR "Botulinum toxin" OR "Botulinum Toxin A" OR "Botulinum Neurotoxin" OR "BoNTA" OR "Botox injection" ) )

*Date of search: November 2024*

*Search results: 133*

*No limitations applied*

***WEB OF SCIENCE***

ALL ( ( "Posterior urethral valves" OR "Posterior urethral valve" OR "PUVs" OR "PUV" OR "Congenital obstructing posterior urethral membrane" OR "COPUM" ) AND ( "Valve Ablation" OR "Urethral valve ablation" OR "Primary valve ablation" OR "Posterior urethral valve ablation" OR "Valve Resection" OR "Endoscopic Valve Incision" OR "Transurethral Valve Ablation" OR "Primary Valve Incision" OR "Endoscopic Ablation " OR "Valve Fulguration" ) AND ( "Bladder Neck Incision" OR "BNI" OR "B.N.I." OR "Bladder-neck incision" OR "Bladder Neck Incisions" OR "Transurethral Incision" OR "Incisional Techniques" OR "Posterior Urethral Valve Incisions" OR "Alpha adrenergic blocker" OR "Alpha-blockers" OR "Alpha-1 blockers" OR "Alpha-1 adrenergic blockers" OR "α-1 Adrenergic Blockers" OR "Anticholinergics" OR "Anticholinergic" OR "Oxybutynin " OR "Oxybutinin" OR "Botulinum A" OR "Onabotulinumtoxin A" OR "Botulinum toxin" OR "Botulinum Toxin A" OR "Botulinum Neurotoxin" OR "BoNTA" OR "Botox injection" ) )

*Date of search: November 2024*

*Search results: 21*

*No limitations applied*
